# Occurrence of *Leishmania* spp. DNA and specific antibodies in dogs from Acre State, Rio Branco, Brazil

**DOI:** 10.1590/S1984-29612024072

**Published:** 2024-12-02

**Authors:** Gleice Kelly Carvalho Bento, Leticia Gomes Zanfagnini, Marcia Dalastra Laurenti, Thayse Yumie Tomokane, Vania Lucia Ribeiro da Matta, Soraia Figueiredo Souza, Acácio Duarte Pacheco

**Affiliations:** 1 Centro de Ciências Biológicas e da Natureza, Universidade Federal do Acre - UFAC, Rio Branco, AC, Brasil; 2 Departamento de Moléstias Infecciosas, Faculdade de Medicina, Universidade de São Paulo – USP, São Paulo, SP, Brasil; 3 Setor de Ciências Agrárias, Universidade Federal do Paraná – UFPR, Curitiba, PR, Brasil

**Keywords:** Dogs, DPP, ELISA, *Leishmania* spp, Cães, DPP, ELISA, *Leishmania* spp

## Abstract

Canine leishmaniasis is a parasitic disease whose agents are transmitted through the bites of infected phlebotomine sand flies. This disease is endemic in tropical and subtropical regions, including Brazil. However, information on its prevalence in dogs in some Brazilian states remains limited. This study aimed to assess the seroprevalence of canine leishmaniasis in Rio Branco, Brazil. Blood samples were collected from 375 dogs aged > 6 months. Two distinct serological methods, dual path platform test (DPP) and enzyme linked immunosorbent assay (ELISA), were used to investigate the occurrence of anti-*Leishmania* spp. antibodies. The results showed a seroprevalence of 38.1%, indicating that the disease occurred in this region. Blood samples considered positive in at least one of the serological methods were subjected to conventional polymerase chain reaction (PCR), which confirmed the presence of infection in 28.3% (106/375) of the total samples. This is the first study to provide detailed information on the seroprevalence of canine leishmaniasis in dogs in Rio Branco, highlighting the importance of disease surveillance and control. Effective actions, such as education campaigns on sand fly prevention and control measures, are necessary to reduce the occurrence of canine and human leishmaniasis in cities.

## Resumo

A leishmaniose canina é uma doença transmitida pela picada de flebotomíneos infectados. A doença é endêmica em várias regiões tropicais e subtropicais, incluindo o Brasil. No entanto, informações sobre a prevalência da doença em cães em muitos estados brasileiros são limitadas. Este estudo foi realizado para avaliar a soroprevalência de leishmaniose canina em Rio Branco, capital do Estado do Acre. Para isso, amostras de sangue foram coletadas de 375 cães com idades superiores a 6 meses. Para ocorrência de anticorpos anti-*Leishmania* spp, foram utilizados dois métodos sorológicos distintos, o ELISA e o DPP®. Os resultados mostraram uma soroprevalência de leishmaniose canina de 38,1%, indicando que a doença é endêmica na região. As amostras de sangue, consideradas reagentes em pelo menos um dos métodos sorológicos, foram submetidas à Reação em cadeia da polimerase convencional (PCR) e confirmaram a presença de infecção em 28,3% (106/375) desse total. Este estudo é o primeiro a fornecer informações detalhadas sobre a soroprevalência de leishmaniose canina em cães em Rio Branco, destacando a importância da vigilância e controle da doença na região. Ações efetivas, como campanhas de educação sobre medidas de prevenção e controle de flebotomíneos, são necessárias para retardar a dispersão da enfermidade na cidade.

## Introduction

Leishmaniasis, a disease caused by protozoa of the genus *Leishmania,* is considered a neglected tropical disease by the World Health Organization (WHO, 2021). Dogs play a crucial role in the zoonotic cycle of visceral leishmaniasis (VL) and are considered the most important animal reservoir.

However, discrepancies exist in the literature regardind the role of dogs in transmitting the cutaneous form of the disease, especially in regions where other potential reservoir hosts have not been identified (Marcondes & Day, 2019). Nevertheless, *Leishmania amazonensis* has been detected on the skin of these animals (Dantas-Torres et al., 2012; Estevam et al., 2022). This highlights the importance of definitive diagnosis in canine cases, particularly those with subclinical infections. Diagnosis can be achieved through specific tests like parasitological, serological, and/or molecular methods (Marcondes & Day, 2019).

Considering the varied clinical presentations in canine cases, a definitive diagnosis of infection should be made through specific tests, such as parasitological, serological, and/or molecular tests, mainly in subclinically infected dogs (Marcondes & Day, 2019). Among serological methods, the Ministry of Health recommends the dual-path platform (DPP) rapid test for initial screening. The indirect enzyme-linked immunosorbent assay (ELISA) can then confirm cases of canine VL, particularly when more specific tests like polymerase chain reaction (PCR) are unavailable (Marcondes & Day, 2019).

Human cutaneous leishmaniasis is endemic in Acre, Brazil, with an intra/peridomiciliary transmission profile observed in the microregions of Cruzeiro do Sul, Rio Branco, and Tarauacá (Melchior et al., 2017). Confirmed cases exist in both humans and dogs (Ávila, 2018). However, no studies have yet evaluated seroprevalence, *Leishmania* species involved, or identified other potential reservoir hosts in these areas.

Seroprevalence studies are crucial for understanding the occurrence of infection and disease in a region. This knowledge guides preventive and control measures, especially when early identification of potential hosts is possible (Selim et al., 2021; Morales-Yuste et al., 2022). Notably, human cases have been reported in urban areas of Acre. Therefore, this study aimed to assess the presence of anti-*Leishmania* antibodies in dogs from the Municipality of Rio Branco, Acre.

## Material and Methods

Rio Branco’s Department of Zoonosis Control estimates a dog population of approximately 48,289. A non-probability convenience sampling design was chosen with a 95% confidence interval, 5% absolute precision, and an expected prevalence of 50%. This approach allows estimation of the prevalence in a large population with established confidence limits based on the sample size (Levy & Lemeshow, 2005). Therefore, a sample size of approximately 384 was targeted.

A total of 375 blood samples were collected from dogs at various locations: Rio Branco Veterinary Clinics, Department of Zoonosis Control, University Veterinary Hospital of the Federal University of Acre, and temporary shelters run by animal protection non-governmental organizations. All dogs were over six months old, and both sexes were included.

For serological tests, blood samples were collected via puncture of the jugular or cephalic veins after proper antiseptic preparation and physical restraint. Whole blood samples were stored in clotting-free tubes and centrifuged for 5 minutes at 5000 rotations per minute (rpm) to obtain the serum. They were subsequently labeled, stored in 1,5 mL microtubes, and kept at −20°C until processing.

The DPP^®^ rapid immunochromatographic test was performed according to the manufacturer's recommendations. The ELISA was performed as previously described (Pacheco et al., 2013). Serum samples from dogs from an area where *Leishmania* spp. is endemic were used as positive controls. Samples from dogs from an area where canine leishmaniasis is non-endemic were used as negative controls. The cutoff point was determined based on the mean and two standard deviations of the optical density (OD) readings of the negative controls. Animals with results above the cutoff point were considered reactive.

The DNA extraction and purification were performed the PCR were performed using silica columns and the NucleoSpin® Tissue, following the manufacturer's instructions. The DNA was subsequently stored at −20°C until it was ready for use.

For the PCR procedure, the primer oligonucleotides previously described, namely, LEISH-1 (5ʹ-AACTTTTCTGGTCCTCCGGGTAG-3ʹ) and LEISH-2 (5ʹACCCCCAGTTTCCCGCC-3ʹ), were used (Francino et al., 2006), resulting in a 120 bp fragment of *Leishmania* kinetoplast DNA (kDNA). Briefly, 10 μL of GoTaq Green Master Mix 2x (Promega, USA), 4.8 μL of ultrapure water, 0.5 μL of each primer at 10 μM, 0.2 μL of 5 M tetramethylammonium chloride (TMAC), and 4 μL of DNA from each previously extracted sample were used. The samples were amplified in a thermocycler Applied Biosystems®, with an initial DNA denaturation step at 95 °C for 5 minutes, followed by 35 cycles at 95 °C (double-stranded DNA denaturation) for 15 seconds, 60 °C (primer annealing) for 20 seconds, and 72°C (DNA polymerase-mediated new strand synthesis) for 1 min. A final cycle of 72°C for 10 min was performed.

For molecular analysis, a 2% agarose gel (100 mL) was prepared in Tris/Acetic Acid/EDTA buffer with 5 μL of GelRed nucleic acid stain (Biotium, USA) added. Then, 5 μL of amplified samples and a molecular weight marker (KAPA Universal Ladder, USA) were loaded into each well. Electrophoresis was performed at 100 V for three hours. The gel was then photographed under ultraviolet light using a CareStream Photodocumentation System (China) to visualize bands generated by restriction enzyme digestion.

Samples negative in the initial PCR with LEISH1/LEISH-2 primers, even after re-amplification (nested-PCR), underwent additional PCR targeting mammalian beta-actin. Specific primers (forward: 5ʹGACAGGATGCAGAAGGAGAT-3ʹ, reverse: 5ʹTTGCTGATCCACATCTGCTG-3ʹ) were used (Francino et al., 2006). This ensured negative results from the conventional PCR reflected true negatives and were not due to degraded DNA or PCR inhibitors. The cycling conditions remained the same as those used for the LEISH-1/LEISH-2 primers.

To analyze and compare the obtained optical densities, the samples were grouped as follows: G1, seropositive using the ELISA test; G2: seropositive using the DPP test; G3: seropositive using both serological tests; and G4: samples not reactive in either serological test. Subsequently, the Kruskal–Walli’s test was used to determine statistically significant differences among the groups.

Agreement between the ELISA and DPP tests was assessed using the Kappa coefficient. Interpretation of concordance was based on established Kappa values using the Statistical Analysis System software. The Fisher exact test (*p* <0.05) was employed to calculate the relative and absolute frequencies of results from each test (ELISA and DPP). Additionally, it evaluated potential associations between variables like age, sex, collection location, and clinical signs.

## Results

Most of the sample population originated from private clinics in Rio Branco (41.3%), followed by animals rescued by NGOs (33.86%), clinical care at the Teaching and Research Unit in Veterinary Medicine at the Federal University of Acre (13.3%), and animals from the Rio Branco Zoonosis Department (11.4%).

The age of the animals ranged from 7 months to 12 years, with 75.20% being adults (between 1 and 6 years old), 19.20% being older than 7 years, and only 5.60% being less than one year old. Females were predominant, representing 63.20% (237/375) of the study population.

Of the 375 dogs, 143 (38.13%) tested positive for anti-*Leishmania* spp. antibodies using both serological methods. The ELISA test identified a seroprevalence of 19.68% (74/375), while the DPP test showed reactivity in 18.40% (69/375) of the samples. Only 5% (19/375) of dogs reacted positively to both tests simultaneously. Notably, the agreement between the serological tests was considered weak (kappa = 0.07). Among ELISA-positive animals, 52.7% (39/74) displayed clinical signs, while the DPP test identified clinical signs in 48.57% (34/69) of reactive animals.

The most frequent clinical manifestations observed were hypotrichosis (50%), followed by cachexia (36%), localized alopecia (26%), silvery scaling (22%), generalized alopecia (22%), nasodigital hyperkeratosis (10%), periocular alopecia (9%), skin lesions (9%), ulcers, onychogryphosis (8%), weight loss (6%), uveitis (6%), lymphadenopathy (5%), and exfoliative dermatitis (3%). Less frequent abnormalities (19.2%) included ocular discharge, cutaneous erythema, dehydration, pale mucous membranes, presence of ectoparasites, seborrhea, cutaneous hyperpigmentation, otopathy, keratoconjunctivitis, moist dermatitis, corneal ulcers, hyperthermia, cutaneous nodules, conjunctivitis, epistaxis, rhinitis, melena, vomiting, and pigmentary keratitis.

Statistical analysis revealed no significant differences in sex (*p* = 0.67), age (*p* = 0.49), or collection location (*p* = 0.137) when evaluating these variables in relation to the serological test results.

When the means of the optical densities (OD) were assessed according to the association of the serological methods used, it was possible to observe that animals considered simultaneously reactive in both methods exhibited significantly higher OD values than those that were reactive only using DPP or those considered non-reactive (Table 1).

**Table 1:** Means of optical densities (OD) and standard deviations found in seropositive animals, followed by analysis through the Kruskal-Wallis test at a 5% significance level (p<0.001).

	**OD Avarage**	**Standart deviation**
DPP and ELISA	0.417^a^	±0.2653
ELISA	0.314^a^	±0.199
DPP	0.164^b^	±0.2051
No reagent	0.069^c^	±0.0486

**Figure 1:**
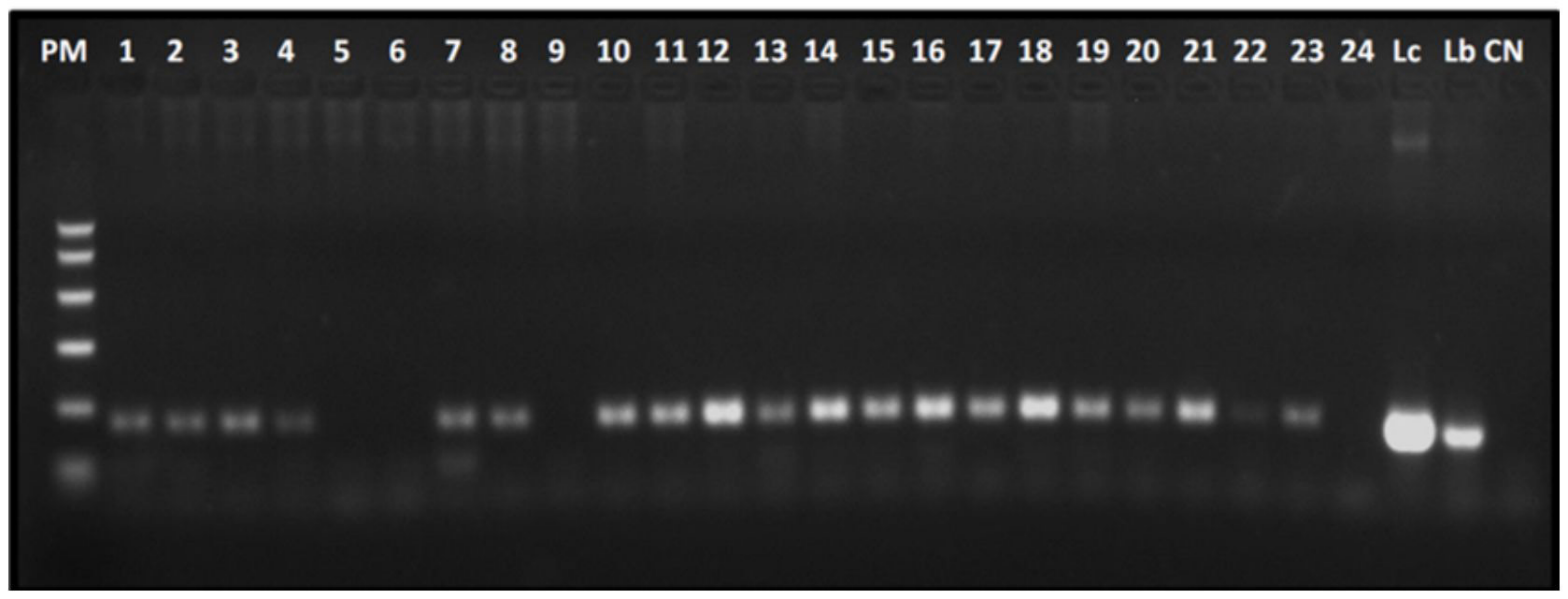
Conventional PCR with LEISH-1 and LEISH-2 primers: 2% agarose gel electrophoresis revealing amplification products of 120 bp kDNA fragments from *Leishmania* sp. in 24 blood samples from dogs from the municipality of Rio Branco, Acre. PM: Molecular weight marker at 50, 150, 300, 500, 750, and 1000 bp. Positive controls: Lc *Leishmania (Leishmania) infantum chagasi* (MHOM/BR/72/LD46), Lb *Leishmania (Viannia) braziliensis* (MHOM/BR/1995/M15280), CN: negative control. Samples 5, 6, 9, and 24 were negative.

Whole blood samples from animals considered reactive using at least one of the serological methods were subjected to conventional PCR using the primers LEISH-1 and LEISH-2 to confirm the presence of parasitic DNA. Thus, it was possible to confirm the protozoan DNA after amplification (Figure 1) and reamplification of 106 animals.

Interestingly, PCR analysis suggested higher accuracy for the DPP test compared to ELISA. When evaluating the serological methods individually, PCR confirmed *Leishmania* infection in 78.26% (54/69) of DPP-reactive animals and 70.27% (52/74) of ELISA-reactive animals.

## Discussion and Conclusion

Dogs play a crucial role in the transmission cycle of leishmaniasis. They act as reservoirs for the parasite *Leishmania* spp., contributing to the spread of the disease. *Leishmania infantum*, specifically, is the culprit behind VL. Transmission to humans and other mammals primarily occurs through the bites of previously infected female sandflies. In Brazil, *Lutzomyia longipalpis* and *Lutzomyia* spp. are the main vectors of *L. infantum* (Andrade-Filho et al., 2017).

Studies in established leishmaniasis regions of Brazil have shown varying seroprevalence for *Leishmania* spp. in dogs, ranging from 3.2% to 50.3%. This variation depends on the study location, sample characteristics (type and size), and diagnostic methods used (Marcondes & Day, 2019; Evaristo et al., 2021a; Xhekaj et al., 2023).

High seroprevalence values in Brazil’s Northern region suggest widespread disease occurrence, potentially similar to other states. This aligns with findings in Rondônia, a neighboring state of Acre, where seroprevalence ranged from 5.06% to 29.81% (Ribeiro et al., 2019). Similarly, studies in Pará reported values between 3.42% and 32.6% (Marcondes & Day, 2019).

The study region is known for its abundance of wild reservoirs and vectors (Araujo-Pereira et al., 2020). This focus on wild animals might underestimate the role of dogs in disease transmission. The literature remains divided on the role of dogs, particularly in the cutaneous form of leishmaniasis (Araujo-Pereira et al., 2020).

Diagnosing canine leishmaniasis accurately often involves a combination of three methods: parasitological examinations for direct parasite visualization, molecular tests like PCR, and serological tests (Ribeiro et al., 2019). This study employed two serological screening methods and compared their results with PCR.

Combining diagnostic methods improves the detection rate of infected animals (Evaristo et al., 2021b). In our study, the performance of ELISA and DPP as initial screening tools was crucial. This was evident when comparing results across different animal groups, regardless of whether they reacted positively to only one or both tests. Our findings support the recommendation to use two diagnostic methods, especially when dealing with low antibody titers in patients or situations where confirmatory methods like PCR are unavailable (Evaristo et al., 2021b).

Interestingly, our study observed very similar positivity rates for both ELISA and DPP, which aligns with Ribeiro et al. (2019) who also reported higher sensitivity for this approach. However, Maia et al. (2022) suggests that relying solely on individual serological tests in Brazil may not be sufficient for guiding control interventions focused on canine reservoirs.

Several factors can influence the accuracy of diagnostic tests for canine leishmaniasis. These include the time since infection, disease severity, the dog’s immune response, potential cross-reactions with other diseases, past vaccinations, and even technical errors during testing (Ribeiro et al., 2019; Maia et al., 2022; Pagniez et al., 2023).

For example, dogs with the cutaneous form of leishmaniasis may have a weak humoral immune response. This means they might not produce enough antibodies to be detected by serological tests (Madeira et al., 2006).

Additionally, clinically healthy dogs may have lower antibody levels compared to symptomatic animals. This is because antibody production is often triggered by the development of clinical signs (Teixeira et al., 2019).

Furthermore, the type of antigen used in each serological test can influence the results, especially in areas where leishmaniasis is not yet well-established (Hirschmann et al., 2015). Studies in regions endemic to the visceral form of the disease have shown higher agreement between different diagnostic methods compared to our study. This suggests that using two serological tests in our study aimed to improve the detection rate despite potential limitations.

Another factor to consider, especially in dogs with low antibody titers, is cross-reactivity (Paltrinieri et al., 2010). Unfortunately, we could not confirm this possibility in our study as we did not perform specific tests for these other pathogens.

Our findings indicate that over 38% of the surveyed dog population had antibodies against *Leishmania* spp. This highlights the importance of further epidemiological studies investigating the role of dogs in disease transmission. Additionally, PCR confirmation using samples from Rio Branco dogs is crucial. Implementing routine diagnostic testing will enable better control measures, including dog and human protection, vector control, and public education.
